# From Development to Aging: Dynamic Roles of the Thymic Medulla in T Cell Biology

**DOI:** 10.1111/imr.70118

**Published:** 2026-04-08

**Authors:** Christina Michalaki, Minahil Sharjeel, Jennifer E. Cowan

**Affiliations:** ^1^ Division of Infection and Immunity University College London London UK

**Keywords:** aging, involution, regeneration, thymic epithelial cells, thymic medulla

## Abstract

The thymic medulla is essential for establishing central tolerance, orchestrating the development of a diverse yet self‐tolerant T cell repertoire, and preventing autoimmunity. This process is primarily mediated through interactions between developing thymocytes and antigen‐presenting cells, including thymic epithelial cells (TECs) and dendritic cells (DCs), with additional regulatory contributions from endothelial cells, mesenchymal cells, and macrophages. Despite its critical role, the complexity of late‐stage thymocyte development and the dynamics of their medullary residency remain incompletely understood. Recent advances in single‐cell, epigenomic, and transcriptomic technologies have begun to reveal previously unappreciated layers of cellular and molecular heterogeneity within the thymic medulla throughout life. In this review, we explore how the medulla shapes the fate of both conventional and non‐conventional T cells, examine the diversity of thymocyte populations it supports, and discuss how this specialized microenvironment adapts during aging and regeneration.

## 1 Introduction

The thymus is the primary site of T cell development. Early thymocyte development occurs within the cortex where hematopoietic progenitors, entering at the cortico‐medullary junction (CMJ), commit to a T cell lineage and undergo T cell receptor (TCR) rearrangement. If successful at rearranging a functional TCR that can recognize an MHC molecule presenting a small peptide fragment (known as a peptide–MHC), the developing T cell receives a TCR signal and is positively selected, after which it commits to a CD4 or CD8 T cell fate and migrates from the thymic cortex into the medulla. Within the medulla, CD4 single positive (SP4) or CD8 single positive (SP8) cells undergo a stringent selection process, where they are screened for self‐reactivity, and any developing T cells with potential specificity to self are depleted, in a process known as negative selection. This is essential for the establishment of central tolerance. Furthermore, the thymic medulla fosters the generation of non‐conventional T cell lineages, such as T‐regulatory (Treg) cells, which are also essential in establishing tolerance [1]. Developing T cells that successfully rearrange a functional TCR and are deemed not to be self‐reactive, exit into the periphery as a recent thymic immigrant (RTE) from the CMJ and home to secondary lymphoid tissue [2].

This multi‐step process and stringent migratory pathway is essential for successful T cell development and is heavily orchestrated by the resident thymic epithelial cells (TECs). TECs can be separated into two major subsets according to their spatial location: cortical TECs (cTECs) and medullary TECs (mTECs). cTECs are essential for the earlier stages of T cell development, whilst mTECs play a pivotal role in the later stages of thymocyte development. TECs are highly specialized and uniquely adapted to enable distinct functional roles by presenting distinct self‐antigens to developing T cells, along with expressing unique chemokines that mediate the migration of thymocytes from the cortex to the medulla [2].

The TCR restricts T cells to recognizing antigens presented within an MHC molecule. Random gene rearrangements during TCR formation generate an extensive repertoire, maximizing the ability to detect diverse foreign antigens. However, this stochastic process also produces non‐functional TCRs or TCRs with the potential to recognize self‐antigens. TECs play a central role in testing TCR functionality: weak engagement of the TCR with self‐peptide MHC complexes provides survival signals, allowing thymocytes to undergo positive selection. In contrast, strong TCR signals from recognition of self‐antigens trigger either deletion through negative selection or diversion into a tolerogenic fate, such as the Treg lineage via agonist selection [2]. This careful discrimination between self and non‐self ensures the generation of a self‐tolerant T cell pool and prevents a breakdown of central tolerance leading to autoimmunity [3]. Thymic selection is extremely stringent, eliminating the vast majority of thymocytes and allowing only one to three % to survive and populate the peripheral T cell pool [4, 5].

Both positive and negative selection can be mediated by cTECs, whereas medullary mTECs primarily drive negative selection and agonist selection. Each TEC subset presents a distinct repertoire of self‐antigens to developing thymocytes. cTECs generate self‐peptides through specialized antigen‐processing machinery that produces peptide repertoires enriched for low‐affinity TCR ligands. This includes the proteasome, which degrades ubiquitinated proteins into peptide fragments for surface presentation. Notably, cTECs uniquely and constitutively express the thymoproteasome subunit β5t, encoded by *Psmb11*, which is essential for efficient positive selection of CD8 T cells. Loss of β5t in mice selectively reduces SP8 thymocytes and alters their TCR repertoire [6]. In contrast, mTECs present a broad array of self‐peptides to recapitulate nearly all tissues of the body. This comprehensive representation of ‘whole self’ is achieved through promiscuous gene expression, leading to the synthesis of thousands of tissue‐restricted antigens, a defining and tightly regulated feature of mTECs controlled by the transcriptional regulator Autoimmune Regulator (AIRE), as well as other mechanisms. mTECs can present these antigens directly to SP thymocytes or transfer them to other antigen presenting cells, such as DCs, for cross presentation [7]. Ordered co‐expression of tissue restricted antigens by mTECs, together with their random spatial distribution within the medulla, is thought to optimize encounters with developing thymocytes, although the mechanisms governing this organization remain unclear [8].

Recent advances in single‐cell epigenomic and transcriptomic analyses have revealed a remarkable degree of heterogeneity within the TEC population, particularly among medullary TECs [3, 9]. These discoveries are beginning to uncover the specialized and distinct functions of previously unappreciated TEC states. However, despite this progress, our fundamental understanding of how TECs develop, are maintained, and adapt across the lifespan remains incomplete. In this review, we examine the current framework of TEC biology in T cell development, with a specific focus on the thymic medulla and mTECs. We first examine the mechanisms that regulate the migration of SP thymocytes from the cortex into the medulla following positive selection and consider how cues encountered during medullary residency influence their differentiation into non‐conventional T cell lineages, including Treg and invariant natural killer T cells (iNKT cells). Building on this intrathymic framework, we then address the dynamic exchange between the thymus and the periphery by examining the recirculation of mature T cells back to the medulla. We extend discussions to the medullary environment and function across defined stages of life, with particular emphasis on age‐associated thymic involution and acute involution following injury. Finally, we integrate these concepts by discussing how advances in our understanding of medullary function are informing strategies for thymic rejuvenation, with potential implications for improving immune competence.

## 2 The Thymic Medulla in Determining T Cell Fate

### 2.1 Entry of Positively Selected Thymocytes Into the Medulla

The migration of positively selected thymocytes from the cortex to the medulla is a critical checkpoint in T cell education essential in the establishment of central tolerance [10, 11]. The positive selection of Double Positive (DP) thymocytes through recognition of self‐peptide MHC complexes on cTECs initiates their exit from the cortex and entry into the medulla. This medullary positioning of SP thymocytes is tightly regulated by chemokine gradients, and disruption of this migratory program has profound consequences for late‐stage thymocyte differentiation and medullary formation [10]. Among the chemokine pathways involved, CCR7 and its ligand CCL21 are the principal regulators of medullary entry [12, 13, 14]. TCR engagement during positive selection induces CCR7 upregulation on thymocytes, promoting their directed migration across the CMJ into a CCR7 ligand enriched medullary environment [15, 16]. In mice lacking CCR7 or its ligands, SP thymocytes accumulate within the cortex and medullary areas are reduced in size and appear fragmented. Some SP thymocytes are present within the medulla, suggesting a possible role for additional chemokine receptors [15]. Although mature SP thymocytes can exit the thymus directly from the cortex, this bypass compromises negative selection against tissue‐restricted antigens (TRAs) and predisposes mice to autoimmunity [10, 17]. Premature CCR7 expression is sufficient to redirect DP thymocytes into the medulla [18]. Furthermore, CCR7 mediated medullary migration is important for the differentiation of non‐conventional αβ T cell lineages such as iNKT‐cells [1, 19]. This CCR7‐dependent migration must coincide with the movement of newly positively selected thymocytes away from a CCL25 enriched cortex, despite their high expression of its receptor CCR9. This process is directed by PlexinD1 which, upon activation, represses CCL25 chemokine signaling and prevents thymocyte retention in the cortex [20].

In addition to CCR7 and PlexinD1, it has been speculated that other chemokines mediate this critical migration of SP thymocytes, including CCR4. This is due to this chemokine receptor being expressed by the earliest positively selected thymocytes, prior to CCR7 upregulation, and its ligands, CCL17 and CCL22, being predominantly expressed by thymic DCs residing in the medulla [19, 21, 22, 23]. Regardless of this interesting expression pattern, we found that CCR4 is dispensable for the migration and development of SP thymocytes into the medulla, even in combination with CCR7 deficiency [19]. Others have found, however, using thymocyte‐seeded thymic slices, that CCR4‐deficient thymocytes accumulate less efficiently in the medulla and that their interactions with medullary DCs are perturbed, thereby altering central tolerance [23, 24]. Together, these studies highlight chemokine‐guided migration as a central determinant of medullary residency and tolerance induction. While CCR7 and PlexinD1 act as the dominant drivers of cortical to medullary thymocyte relocation, additional chemokine receptors such as CCR4 fine‐tune intramedullary positioning and cellular interactions required for effective central tolerance. As combined CCR7 and CCR4 deficiency does not completely abrogate SP migration into the medulla, this suggests that additional, as yet unidentified, chemokines and their receptors may contribute to mediating this essential migratory step.

### 2.2 Conventional and Non‐Conventional T Cell Differentiation in the Medulla

Once within the medulla, SP thymocytes are estimated to reside for approximately five days before exiting to the periphery as naïve T cells in the mouse [25]. During this residency, SP thymocytes undergo a series of post‐positive selection events, the most notable of which is negative selection. In addition, they progress through several phenotypically and functionally distinct maturation stages and commit to either conventional or non‐conventional Treg lineages [1, 26]. This lineage commitment is mediated by the medulla and antigen‐presenting cells (APC), chiefly mTECs. Disruptions to mTECs can result in a severe hindrance to the development of Treg, yet, surprisingly, conventional T cells can mature in their absence, although at the expense of tolerance [11, 27, 28].

### 2.3 Conventional T Cell Differentiation

**FIGURE 1:**
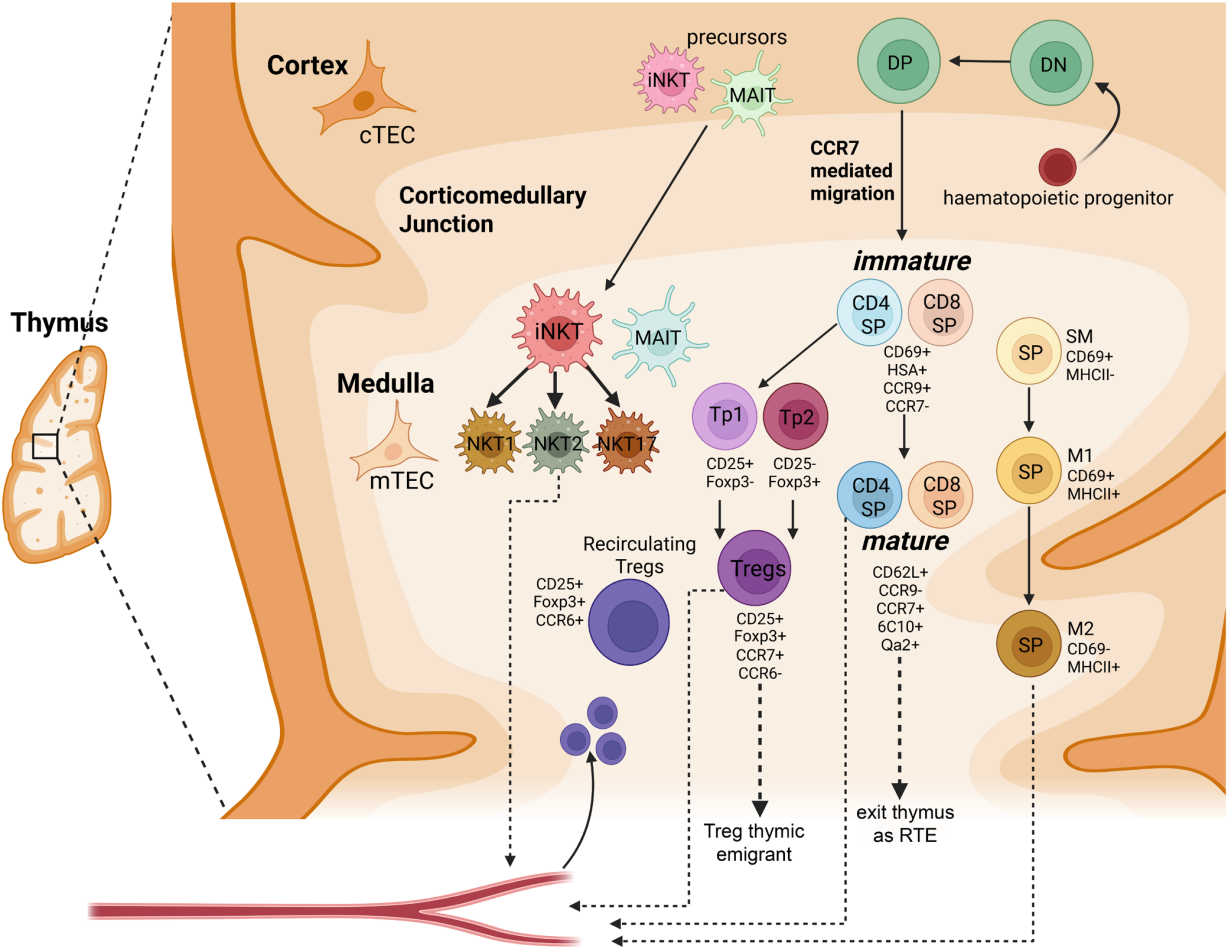
Development of conventional and non‐conventional lineages in the thymic medulla. The thymus is organized into cortical and medullary compartments that support sequential stages of T cell development. Hematopoietic stem cells enter at the cortico‐medullary junction into the cortex and progress through double‐negative (DN) and double‐positive (DP) stages before positive selection. Positively selected thymocytes differentiate into CD4^+^ or CD8^+^ single‐positive (SP) cells and migrate to the medulla in a CCR7‐dependent manner. Immature SP thymocytes (CD69^+^HSA^+^CCR9^+^CCR7^−^) undergo further maturation (CD62L^+^CCR9^−^CCR7^+^6C10^+^Qa2^+^) prior to thymic egress as recent thymic emigrants (RTEs). SP thymocytes can be further subdivided into three subsets: Semi‐mature (SM) (CD69^+^ MHC Class I^−^), Mature 1 (M1) (CD69^+^ MHC Class I^+^), and Mature 2 (M2) (CD69^−^ MHC Class I^+^). CD4^+^ SP thymocytes can also generate regulatory T cells (Tregs) via precursor populations (Tp1: CD25^+^Foxp3^−^; Tp2: CD25^−^Foxp3^+^). Mature thymic Tregs (CD25^+^Foxp3^+^CCR7^+^CCR6^−^) exit the thymus, while recirculating Tregs (CD25^+^Foxp3^+^CCR6^+^) may re‐enter from the periphery. The medulla also supports the development of innate‐like T cells, including invariant natural killer T (iNKT) cells, which differentiate into NKT1, NKT2, and NKT17 subsets, as well as mucosal‐associated invariant T (MAIT) cells. Cortical and medullary thymic epithelial cells (cTECs and mTECs) provide the stromal niches that support selection and lineage diversification.

The major stages of conventional SP4 and SP8 thymocyte maturation can be defined by characteristic surface marker changes (Figure 1). These include loss of heat stable antigen (HSA) and CD69, together with acquisition of CD62L and Qa2 as cells mature [25, 27]. Further subdivision is possible based on chemokine receptor expression, with progressive downregulation of CCR9 and CCR4 and upregulation of CCR7 during maturation [22, 28]. Expression of the carbohydrate epitope 6C10 also changes markedly and, in combination with CD69 and Qa2, separates SP thymocytes into four subsets that show stepwise decreases in 6C10 with increasing maturation [27]. More recently, stages of SP differentiation have been classified into three subsets: semi‐mature (SM), mature 1 (M1), and mature 2 (M2), based on functional competence, including cytokine licensing. M1 can proliferate following TCR stimulation, while M2 produce cytokines and are competent for emigration [29]. Both NFκB and tonic interferon signaling contribute to terminal maturation and are required for thymocyte proliferation and thymic egress [29]. Moreover, the most mature conventional SP thymocytes can be identified by their surface expression of the Sphingosine‐1‐phosphate S1P (1) receptor, essential for their egress from the thymus into the blood [30]. Together, these complementary classification schemes provide a detailed framework for understanding conventional T cell developmental stages. After exiting the thymus, recent thymic emigrants (RTEs) continue their maturation in the periphery [31].

### 2.4 T Regulatory Cell Differentiation

During thymocyte development in the medulla, SP4 can differentiate into Treg cells upon strong TCR stimulation (Figure 1). Treg are an important cell type in maintaining immune homeostasis. Treg that arise in the thymus are termed thymically derived Treg, or tTreg, and are distinct from those induced in peripheral tissues, known as peripheral derived Treg, or pTreg [32]. There are two distinct developmental pathways of tTreg; CD25^+^Foxp3^−^ Treg precursor 1 (Tp1) and CD25^−^Foxp3^+^ Treg precursor 2 (Tp2) [33, 34]. These distinct Treg progenitor subsets are thought to contribute to the tTreg pool comparatively but have distinct developmental properties [33]. High TCR stimulation can cause developing SP4 to upregulate CD25, and other components of the proximal interleukin 2 (IL‐2) signaling pathway [35]. This enables the CD25^+^ Foxp3^−^ Treg precursors to compete for cytokine‐mediated induction of Foxp3 expression to drive Treg differentiation, in a STAT5‐dependent process [36, 37, 38]. Alternatively, high affinity TCR engagement can initiate Foxp3 transcription. Foxp3^+^CD25^−^ Treg progenitor cells also require common γ‐chain cytokine signals as it is vital for their survival and differentiation into mature tTreg because Foxp3 expression is lethal without cytokine signals [34]. The timing and the duration of agonist signaling also plays a role in SP4 developmental fates [35], along with disrupted versus persistent TCR signaling [39]. Although both progenitor subsets follow this shared two‐step developmental process, they possess distinct TCR repertoires and differ in their affinity for self‐antigen. They also engage divergent signaling pathways during differentiation and respond differently to stromal cell interactions and cytokines. Moreover, these two progenitor populations are thought to play distinct roles in protecting against autoimmunity [33].

Interestingly, we found the thymic medulla has a differential requirement for the development of CD4 conventional versus tTreg lineages. Later stages of conventional SP development can occur independent of the medulla. In a thymic environment with severe defects in medullary formation, immature CD69^+^ Qa2^−^ SP thymocytes can mature into CD69^−^ Qa2^+^ SP. Moreover, this maturational step for SP4 can occur extrathymically, if the medulla is bypassed completely, following intravenous transfer into the periphery. Unlike conventional T cells, the development of tTreg cells depends on the medulla, where mTECs support the formation of Foxp3^−^CD25^+^ precursors [28]. To investigate this differential requirement for medullary dependence, we performed kidney capsule transplants of thymic organ cultures [28, 40]. RelB is a subunit of the NF‐kappa B complex and is a critical transcriptional factor for mTEC development [41]. Abnormalities in mice with targeted disruption of the RelB gene result in a severe block in mTEC development, along with altered DC subsets, and consequently multiorgan inflammation [42, 43]. RelB‐deficient embryos were grafted under the kidney capsule of wild‐type (WT) adult hosts, enabling WT thymocyte development to be assessed without indirect effects of constitutive RelB‐deficiency systemically. Our findings revealed that conventional SP4 and SP8 can continue their maturation in the absence of RelB‐dependent mTECs, in contrast to the development of Foxp3^−^CD25^+^ Treg precursors and their Foxp3^+^CD25^+^ Treg progeny which were severely impaired within the grafts [28]. The grafting of RelB‐deficient TECs into athymic nude hosts lacking endogenous T cells including Tregs resulted in autoimmunity associated with a significant reduction in Treg development [28], and Foxn1‐mediated conditional deletion of RelB in all TECs caused a selective loss of mTECs and led to spontaneous autoimmune lesions across multiple organs, together with the production of autoantibodies [44]. Our findings collectively confirm the essential role of mTECs in enforcing central tolerance [28, 44].

Although defects in RelB signaling cause particularly severe disruptions in mTEC development, more subtle alterations within specific mTEC subsets or thymic DCs can also compromise tTreg differentiation [45]. DCs also play an important role in tTreg generation [46]. The absence of CD70 ligands on mTECs and DCs reduces CD27 stimulation in developing Treg cells, thereby impairing their maturation [47]. In addition, tTreg differentiation depends on co‐stimulation through CD28 [48]. Although most studies have focused on IL‐2, both interleukin 7 (IL‐7) and interleukin 15 (IL‐15) also contribute centrally to tTreg differentiation [35], whereas the inflammatory cytokine interleukin 1 (IL‐1) can fine‐tune this process, with elevated IL‐1β levels blocking intrathymic Treg development at the transition between precursor and mature stages [49]. Moreover, recent findings have identified a role for the lymphotoxin axis as an inhibitory checkpoint for Foxp3^+^ CD25^−^ Treg precursor to develop into Foxp3^+^ CD25+ Treg, by limiting interleukin 4 (IL‐4) availability [50].

Aire‐expressing mTECs also play a role in tTreg development. Initial investigations of individuals with autoimmune polyendocrinopathy candidiasis ectodermal dysplasia (APECED), a disease resulting from mutations in AIRE, revealed reduced Treg numbers together with changes in the diversity and composition of their TCR repertoires [51]. In mice, however, Aire deficiency does not result in altered Treg numbers [52, 53]. Nevertheless, Aire‐dependent regulation of mTECs promotes production of the chemokine XCL1, which recruits XCR1‐expressing thymic DCs into the medulla [54]. These DCs are important for capturing and presenting Aire‐dependent antigens during tTreg development [46].

Together, our findings and those of others highlight the central role of the thymic medulla and its highly specialized cellular networks in Treg generation. Even subtle perturbations in antigen presentation, co‐stimulatory signaling, or cytokine availability within this niche can profoundly impact this non‐conventional lineage, which is essential for establishing immunological tolerance.

### 2.5 Invariant Natural Killer T Cell Differentiation

Beyond Treg, the thymic medulla also plays a critical role in the development of other non‐conventional T cell lineages, including CD1d restricted iNKT cells (Figure 1). This non‐conventional αβ T cell lineage recognizes glycolipid antigens presented by CD1d molecules [1, 26]. Divergence into the iNKT lineage occurs in the cortex at the DP stage following recognition of CD1d glycolipid complexes expressed by neighboring DP thymocytes [55, 56]. Although iNKT cells are selected in the cortex, there is a dual requirement for both cTECs and mTECs during iNKT cell development in the thymus [57]. Positive selection induces upregulation of CCR7 on iNKT progenitors, which is essential for their migration into medullary regions [19, 58]. Once in the medulla, iNKT cells gain access to interleukin 15 (IL‐15) *trans*‐presentation by TECs through IL‐15Rα, a signal that is required for survival and progression through later developmental stages. This medullary dependence is illustrated by experiments in which RelB‐deficient thymic grafts that lack mTECs show markedly reduced numbers of mature iNKT cells, while administration of soluble IL‐15/IL‐15Rα complexes partially rescues iNKT development [57].

The medullary iNKT cell population is heterogeneous and can be subdivided into three major subsets: NKT1 (PLZF^lo^T‐bet^hi^IFN‐γ^+^), NKT2 (PLZF^hi^T‐bet^lo^IL‐4^+^), and NKT17 (PLZF^intermediate^RORγt^+^IL‐17^+^) cells (reviewed [59]). iNKT‐cell differentiation is shaped by TCR signaling strength, with strong signals promoting NKT2 and NKT17 development, whereas low‐avidity TCRs bias cells toward the NKT1 lineage [60]. Medullary factors are also critical for subset homeostasis: in addition to the required IL‐15 [61], IL‐25 supports NKT2 development and effector function [62, 63]. Thymic tuft cells, a specialized mTEC subset, described in “Adult TEC diversity”, produce IL‐25 and are essential for the development and function of NKT2 cells [62].

Importantly, iNKT cells themselves contribute to shaping the medullary niche. Mice lacking iNKT cells display impaired mTEC development, demonstrating reciprocal crosstalk between this lineage and the thymic microenvironment. Developing iNKT cells express Receptor Activator of NFκB Ligand (RANKL) at early stages, which drives RANK‐dependent differentiation of mTECs and supports the establishment and maintenance of the Aire‐expressing mTEC subsets. These findings highlight the thymic medulla not only as a site in which maturation and lineage fate decisions occur, but also as a dynamic microenvironment actively shaped by the cells it supports [57, 64].

The thymus is important for the development and differentiation of additional non‐conventional lineages, such as major histocompatibility complex related 1 (MR1) restricted MAIT cells and γδ T cells, which commit earlier during the double negative (DN) and DP stages. MAIT cells are specialized T cells that recognize microbial vitamin B metabolite antigens presented by MR1 and play a key role in antimicrobial immunity (reviewed [26]). γδ T cell differentiation is also thymus dependent [1, 26], and a recent study demonstrated that the adult thymic medulla is required to prime IFN‐γ‐producing γδ T effector cells, with CCR7 and its ligand CCL21 playing a critical role in this process [65].

Taken together, these findings highlight the thymic medulla as a highly specialized microenvironment in which even modest alterations in cellular composition or signaling pathways can profoundly influence non‐conventional T cell development and immune homeostasis. Although diverse thymocyte subsets localize within the medulla, it remains unclear whether they are organized into defined niches. Emerging advances in spatial transcriptomic and multiomic technologies offer the opportunity to address this question by mapping gene expression and cellular identity in situ, enabling a more precise definition of medullary niche architecture and its role in shaping conventional and non‐conventional T cell differentiation.

### 2.6 T Cell Recirculation to the Thymic Medulla

The thymic medulla is not only home to newly generated SP thymocytes but also contains T cells that have migrated back into the thymus from the periphery. It has long been reported that peripheral CD4 and CD8 T cells can return to their site of development under steady state conditions [66, 67, 68]. These recirculating T cells were first shown to preferentially localize to the thymic medulla, predominantly display an activated phenotype, and persist there for prolonged periods [66, 69]. Chronic reductions in T cell production have also been shown to alter T cell recruitment back to the thymus. Reduced thymic output results in increased T cell recirculation, with a particular bias toward Treg [70]. Furthermore, following intravenous transfer of T cells, the neonatal thymus appears especially permissive to the entry of mature T cells [71]. However, the mechanisms driving enhanced T cell recirculation to the thymus during lymphopenia or neonatal life remain unclear [67].

### 2.7 Treg Recirculation Back to the Thymus

More recent analyses of recirculating T cells have revealed a pronounced bias toward the Treg lineage. It has been suggested that approximately 40%–50% of Treg cells in the adult thymus may represent peripheral recirculants. Thus, the intrathymic Treg compartment is highly heterogeneous, comprising newly generated thymic Treg cells together with substantial numbers of peripheral Treg migrants [25, 72, 73, 74]. To resolve the origin and composition of this heterogeneous thymic Treg compartment, the Rag2GFP reporter mouse has proven an invaluable tool, providing a molecular timer of thymocyte age [25, 75]. Following positive selection, Rag2 gene expression is terminated, and the approximately 56 h half‐life of GFP enables tracking of post‐positively selected thymocytes as they mature from this developmental stage [25, 31]. T cells that have lost GFP expression are estimated to be at least three weeks past TCR gene rearrangement [31]. Analysis of the intrathymic Treg population in Rag2GFP reporter mice revealed a heterogeneous mixture of Rag2GFP^+^ and Rag2GFP^−^ CD25^+^ Foxp3^+^ Treg cells. The Rag2GFP^+^ subset comprises newly generated, *de novo* Treg cells, whereas the Rag2GFP^−^ population likely represents long‐term thymic residents and/or peripheral Treg cells that have migrated back into the thymus [25, 72, 73]. Notably, Rag2GFP^−^ CD25^+^ Foxp3^+^ Treg cells constitute a substantial proportion of the total thymic Treg pool, enabling the estimation of up to 50% of Treg cells in the adult thymus. Importantly, thymic CD25^+^ Foxp3^−^ and CD25^−^ Foxp3^+^ Treg precursor populations also contain Rag2GFP^−^ subsets, albeit at lower frequencies than Treg cells, which may reflect contamination of these precursor gates by mature recirculating T cells [74]. Furthermore, it has been reported that Rag2GFP^+^ newly generated Treg cells decline with age, whereas the Rag2GFP^−^ Treg population expands in the aging thymus [72, 76]. However, *de novo* Treg generation has been reported to remain stable for the first 8 months of life in the mouse thymus, with only modest reductions observed by 1 year of age [77]. The Rag2GFP model can also be used to identify Rag2GFP^+^ RTEs in the periphery [31].

The inability to distinguish long term thymic resident Treg cells from recirculating Treg cells within the Rag2GFP^−^ pool prompted the use of multiple experimental approaches to determine the origin of this subset. One approach involved intravenous transfer of in vitro activated T cells into WT hosts, demonstrating that both conventional and regulatory T cell lineages can migrate back into the thymus [76]. We further used a kidney capsule transplantation model in which congenically marked embryonic WT thymic lobes were transplanted into WT recipients. This strategy allowed a single wave of donor derived, newly generated T cells to be tracked within host tissues. Recipients were analyzed four to six weeks after transplantation. A small proportion of graft derived T cells was detected in the host spleen, with CD4 T cells, CD8 T cells, and Treg cells present at frequencies comparable to those observed among host cells in the same organ. In contrast, a smaller fraction of graft derived T cells was identified in the host thymus, with a strong skewing toward a CD4 positive phenotype, of which 60% to 80% were Treg cells. Our findings indicate that Treg cells possess a preferential capacity for thymic homing compared with conventional T cells generated within the same graft [74]. However, this enrichment does not exclude the possibility that a proportion of the Rag2GFP^−^ Treg pool in the adult thymus represents long term resident cells rather than recirculating Tregs.

It also remains unclear if the developmental origin of a Treg cell influences its propensity to return to the thymus, as thymic derived and peripherally induced Treg populations cannot be reliably distinguished following their exit from the thymus [78]. Although phenotypical, functional and transcriptional differences have been described between Treg cells residing in lymphoid tissues and those circulating in non‐lymphoid tissues [79, 80], it is not known if Treg cells that re‐enter the thymus acquire distinct phenotypic or functional characteristics. Recirculating Treg cells have been reported to display an activated phenotype, characterized by expression of CD44, CD25, CD103, TIGIT and Nrp1 [72, 76], similar to other T cell populations capable of thymic homing [66]. However, more recent analyses have identified both naïve‐like and memory‐like phenotypes within the peripherally derived thymic Treg pool, based on differential CD44 and CD62L expression [77]. These findings reveal substantial heterogeneity among recirculating Treg cells and indicate that antigen experience is not an absolute requirement for thymic re‐entry. Collectively, this complexity warrants the need to define more precisely the relative contribution of the medulla on *de novo* Treg generation versus peripheral recirculation, and to elucidate the molecular mechanisms that govern Treg trafficking and retention within the thymic medulla.

### 2.8 Mechanisms Mediating Treg Homing Back to the Thymus

The strong bias of Treg homing back to the thymus raises the question of what mechanisms mediate this re‐entry. Adhesion molecules and chemokine gradients have been explored in the broader context of all mature T cells entering the thymus [67], however, mediators have been identified that are specifically involved in recirculating Treg. CXCR4 has been identified as a key mediator of peripheral Treg homing to the thymus, with CXCR4 inhibition reducing recirculating Treg by approximately 50% [76]. CCR7 can also facilitate thymic homing of peripheral Treg [74]. Interestingly, we demonstrated that adult Ccr7 knockout (KO) mice exhibit an expanded intrathymic Treg population [19, 81]. When crossing Ccr7 KO mice with the Rag2GFP model, we revealed that this accumulation is not due to an expansion of newly generated, *de novo* Rag2GFP^+^ CD25^+^ Foxp3^+^ Treg, but rather an increase in the Rag2GFP^−^ fraction. To determine whether this reflects enhanced retention or increased recirculation, we performed kidney capsule transplants of WT embryonic thymic grafts into Ccr7 KO or WT adult hosts. Post‐transplantation, significantly more graft‐derived Treg were detected in the thymus of Ccr7 KO hosts compared to WT controls, whilst graft‐derived peripheral Treg numbers remained similar. Our results indicate that CCR7 deficiency promotes Treg circulation back to the thymus, suggesting a role for CCR7 in limiting thymic re‐entry of peripheral Treg. Notably, CCR7 is not expressed on intrathymic Rag2GFP^−^ CD25^+^ Foxp3^+^ Treg, whereas it is uniformly expressed on newly generated Rag2GFP^+^ CD25^+^ Foxp3^+^ Treg. This pattern suggests an indirect role for CCR7 in limiting the contribution of recirculating Treg, independent of CCR7–CCL19/CCL21 mediated migration [74]. One potential mechanism may involve the known alterations in thymic architecture observed in Ccr7 KO mice, which could indirectly affect the recruitment of recirculating Treg [15]. Further investigation may help clarify the relative contributions of direct and indirect CCR7‐dependent mechanisms and could also identify additional pathways that play important roles in this process.

Rag2GFP^−^ CD25^+^ Foxp3^+^ thymic Treg cells lack Ccr7 expression but instead express high levels of the chemokine receptor CCR6, which is absent on their newly generated Rag2GFP^+^ counterparts. This differential chemokine receptor expression pattern enables the discrimination of *de novo* CCR7^+^ CCR6^−^ Rag2GFP^+^ Treg cells from CCR7^−^ CCR6^+^ Rag2GFP^−^ thymus‐recirculating Treg cells [74]. We crossed the Ccr6 KO mice to Rag2GFP reporter mice and showed no significant alteration in total thymic Treg numbers. However, Ccr6 KO mice exhibited reduced frequencies and absolute numbers of Rag2GFP^−^ CD25^+^ Foxp3^+^ Treg cells, accompanied by an expansion of newly generated Rag2GFP^+^ CD25^+^ Foxp3^+^ Treg cells in the adult thymus. To determine whether this phenotype reflects impaired Treg recirculation rather than altered thymic retention, we transplanted Ccr6 KO embryonic thymic grafts under the kidney capsule of WT adult hosts. Following transplantation, reduced numbers of donor‐derived Ccr6‐deficient Treg cells were detected in the thymus of WT recipients compared with mice receiving WT grafts. In addition, donor‐derived Foxp3^+^ Treg cell numbers in the spleen were also reduced in recipients of Ccr6 KO grafts. Collectively, our data support a role for CCR6 in promoting the migration of peripheral Treg cells back to the thymus and potentially to secondary lymphoid organs [82].

CCL20 is the ligand to CCR6. In the adult mouse thymus, early studies suggested CCL20 is predominantly expressed by mTEC^hi^ cells, defined by high CD80 and MHC class II expression. This subset includes Aire^+^ mTECs [82], which have known roles in influencing expression of intrathymic chemokines such as XCL1 [54] and CCR4/CCR7 ligands [21]. Recent work using single‐cell RNA sequencing identified thymic microfold cells, part of the medullary mimetic cell population (described in section “Adult TEC diversity”), as the principal source of CCL20, and showed that this expression is Aire‐dependent [83]. We investigated Ccl20 mRNA levels in total mTEC^hi^ cells from Aire KO and WT mice and demonstrated a significant reduction in the absence of Aire [82]. We crossed Aire KO mice to the Rag2GFP reporter strain and observed a marked reduction in intrathymic Rag2GFP^−^ CD25^+^ Foxp3^+^ recirculating Treg cells, while *de novo* Rag2GFP^+^ Treg cells were unaffected. Moreover, we performed kidney capsule grafting of WT embryonic thymic grafts into WT or Aire KO hosts and confirmed this reduction was a consequence of altered migration of CCR6^+^ peripheral Treg, with Aire KO recipient thymi having reduced migrant graft‐derived Treg, compared to the WT and altered Treg to T conventional ratios. Importantly, the spleens of both WT and Aire KO mice had comparable graft‐derived Treg, indicating that the reduction in the thymus was not due to limited availability. Together, our findings demonstrate that Aire‐dependent CCL20 expression is required for CCR6^+^ peripheral Treg migration into the thymus, identifying an additional role for Aire in regulating intrathymic chemokine availability and Treg entry [82]. Further investigation by others into the role of Aire positive mTECs in CCR6^+^ recirculating Tregs showed that Aire also regulates their suppressive function. Thus, Aire confers mTECs with the ability to control the restimulation of CCR6^+^ recirculating Tregs in the thymus [84].

Transcriptional and phenotypic profiling of CCR6^+^ Rag2GFP^−^ Treg cells enabled the identification of distinct molecular features of this migratory population, including expression of CD73 [33] and IL‐18 receptor [85]. Adoptive transfer of IL‐18R^−^ and IL‐18R^+^ Treg into host mice revealed that IL‐18R^+^ Treg cells exhibited a greater capacity to recirculate from the periphery to the thymus. To determine whether IL‐18R plays a functional role in thymic homing, IL‐18R‐deficient Treg cells were adoptively transferred into WT mice and showed a reduced ability to home to the thymus compared with their WT counterparts. These findings indicate that IL‐18R is required for peripheral Treg cells to re‐enter the thymus. Furthermore, in vitro IL‐18 stimulation increased CCR6 expression on Treg cells, while having no effect on CCR6 levels in conventional T cells or in IL‐18R‐deficient Treg cells. These data support a role for the IL‐18‐IL‐18R pathway in promoting migration of peripheral Treg cells to the thymus through upregulation of CCR6 [85].

**FIGURE 2:**
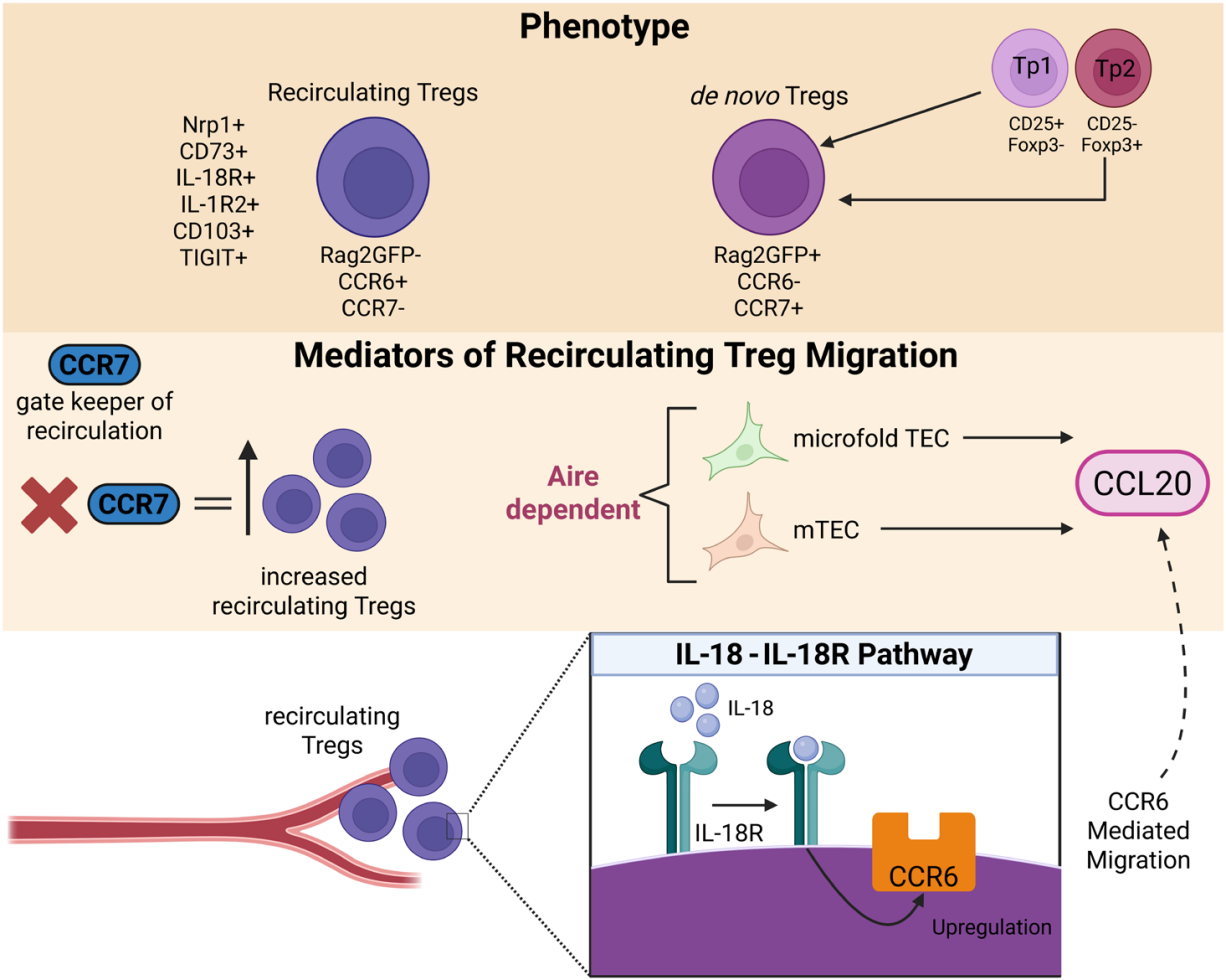
Phenotypic distinctions between newly generated and recirculating Tregs and mechanisms mediating thymic recruitment. Phenotypically, *de novo* Tregs are Rag2GFP^+^ CCR6^−^ CCR7^+^ and arise from Treg precursors (Tp1: CD25^+^ Foxp3^−^ and Tp2: CD25^−^ Foxp3^+^). In contrast, recirculating Tregs are characterized by a Rag2GFP^−^ CCR6^+^ CCR7^−^ phenotype and express additional markers including CD73^+^, IL‐18R^+^, CD103^+^, IL‐1R2^+^, Nrp1^+^, and TIGIT^+^. Medullary thymic epithelial cells (mTECs), including microfold TECs, produce CCL20 in an Aire‐dependent manner. CCR6^+^ recirculating Tregs migrate toward the CCL20 gradient, facilitating their recruitment to the thymus. CCR7 functions as a gatekeeper that restricts the accumulation of recirculating Tregs in the thymus, with loss of CCR7 leading to increased numbers of these cells. In parallel, signaling through the IL‐18–IL‐18R pathway promotes CCR6 upregulation in the periphery, further supporting CCR6 mediated migration of recirculating Tregs to the thymus.

Collectively, our findings and others highlight the thymic medulla as the central site regulating peripheral Treg re‐entry. Chemokine pathways including CXCR4 [76], CCR6 [82] and CCR7 [74] coordinate this process, with the CCR6‐CCL20 axis playing a prominent role, partly driven by Aire‐dependent production of CCL20 by mTEC [78], and potentially microfold cells [83]. In addition, IL‐18 stimulation in the periphery promotes CCR6 upregulation on Treg, facilitating their migration back to the thymus [85]. Our investigations, along with others in Aire KO and Ccr7 KO mice further highlight the importance of medullary organization for successful Treg rehoming, with CCR7 limiting Treg accumulation in the thymus [74], whereas Aire appears to facilitate their entry [82] and suppressive function [84]. The differential markers of recirculating Treg versus *de novo* Treg, along with the key mechanisms governing Treg recruitment, are summarized in Figure 2.

Disruption of the CCR6‐CCL20 axis does not completely abrogate peripheral Treg accumulation, indicating that additional medullary chemokines and adhesion pathways remain to be defined. It also remains unclear whether Treg use thymus‐specific migratory mechanisms or share recruitment programs with other tissues. Notably, CCL20 is known to direct CCR6^+^ Treg to inflammatory sites [86], and tumor microenvironments [87], suggesting potential overlap in trafficking pathways. A recent study identified the CCR6‐CCL20 axis as a driver of Treg recruitment in colorectal cancer. Colorectal cancer samples showed elevated RANKL and RANK expression, which, when overexpressed, enhanced CCL20 production and promoted CCR6‐mediated Treg migration. This consequently boosted tumor stemness and facilitated disease progression [88].

It is also important to consider whether similar mechanisms govern the homing of early T cell precursors to the thymus and the return of mature peripheral T cells. Early T cell progenitors depend on P‐selectin and its ligand, P‐selectin glycoprotein ligand 1 (PSGL‐1), for recruitment from the bone marrow to the thymus [89]. PSGL‐1 is upregulated upon T cell activation and supports migration into non‐lymphoid tissues and inflamed sites [90]. Treg also upregulate PSGL‐1, where it contributes to their suppressive capacity by limiting persistent T cell activation [91]. If PSGL‐1 also contributes to the recruitment of peripheral Treg into the thymus remains an important question.

### 2.9 A Role for Recirculating Tregs

It is still poorly understood why Treg have this preferential bias of recirculating to the thymus, yet this preferential homing suggests that they may serve a functional role [74]. One proposed function of these returning cells is to compete for IL‐2 within the thymic niche, thereby limiting *de novo* Treg differentiation [76, 92]. In this context, the age‐associated accumulation of recirculating Treg could contribute to the decline in thymic Treg production observed with aging [76]. Complementary reports indicate that resident thymic Treg populations compete for a limited IL‐2 supply, providing a negative feedback mechanism for new Treg generation [93]. However, this hypothesis is challenged by the observation that the accumulation of recirculating Treg occurs much earlier in adulthood than the reduction in *de novo* Treg production [77]. Furthermore, in Tnfrsf11b KO mice, which lack the soluble mediator Osteoprotegerin (OPG) which acts as a decoy receptor of RANKL and inhibits RANK‐mediated effects, increased mTEC numbers and increased peripheral Treg recirculation back to the thymus are observed. However, this does not affect the generation of *de novo* Rag2GFP^+^ Foxp3^+^ Treg cells [73].

Migratory Tregs may have roles similar to other cell subsets that home to the thymus, such as DCs, which are known to carry bacterially derived antigens from the gut to the thymus to shape microbe‐specific T cell selection. This provides evidence that migratory populations can influence positive selection and TCR repertoire formation [94, 95]. Peripheral Tregs could act as carriers, shuttling antigens into the thymus that are necessary for mediating TCR repertoire selection. This has been suggested for other activated T cells in the periphery that migrate to the thymus [96], and if the same is true for Tregs, it would be important for understanding potential additional roles of Treg in establishing tolerance.

Thymic recirculating Treg may perform roles in the thymus that are analogous to those of tissue resident Treg in other organs, where they contribute to the maintenance of tissue homeostasis [80]. Another proposed function of recirculating Treg is to modulate intrathymic Treg development under inflammatory conditions. A large fraction of Treg migrants in the thymus express IL‐1R2, a decoy receptor for IL‐1. During systemic inflammation, elevated thymic IL‐1 levels would normally inhibit intrathymic Treg development via IL‐1β signaling; however, recirculating IL‐1R2^+^ Treg can quench this signal and preserve Treg development. Supporting this, the addition of IL‐1R2^+^ Treg to reaggregated thymic organ cultures (RTOCs) restored normal tTreg development in cultures treated with exogenous IL‐1β. These findings indicate that recirculating Treg may have distinct functions under steady state versus inflammatory conditions [49].

More recently, recirculating Treg have been implicated in thymic regeneration following acute injury. Treg are well established as mediators of repair and regeneration in multiple tissues [97]. After an acute insult such as sublethal irradiation (SLI), Treg migrate into the thymus, and disruption of this migration impairs thymic recovery. Moreover, adoptive transfer of Treg prior to SLI enhances thymic regeneration. Transcriptional profiling of Rag2GFP^−^ Treg in the thymus following SLI revealed a distinct migratory signature, including high expression of amphiregulin, a factor known to mediate Treg‐dependent regeneration in other tissues [98]. These observations highlight a unique role for recirculating Treg in thymic repair, although they do not explain the preferential Treg bias observed under steady‐state conditions.

Together, these findings suggest that recirculating Tregs are far from passive bystanders. They actively maintain thymic medullary homeostasis, respond dynamically to inflammatory and regenerative cues, and may modulate *de novo* Treg production. This highlights their critical role in the heterogeneity and the functional adaptability of the thymic Treg compartment. We propose that their preferential thymic homing reflects functions beyond simple cellular trafficking. These cells likely help sustain thymic homeostasis, support tissue repair following physiological stress or injury, and reinforce Treg fitness through renewed exposure to self‐antigens. In this way, recirculating Tregs form a dynamic bridge between peripheral immune experience and central tolerance, linking the periphery with ongoing thymic selection. Understanding why and how Tregs return to the thymus could enable manipulation of this population for therapeutic purposes, including modulation of inflammation, tissue regeneration, or even TCR repertoire engineering.

### 2.10 Summary

In summary, the migration of developing thymocytes into the thymic medulla is indispensable for exposing positively selected cells to the specialized cues that drive negative selection and the lineage specification of both conventional and non‐conventional T cells [10, 28]. Although conventional T cells can complete their maturation without medullary residency, doing so compromises central tolerance and undermines the fidelity of the peripheral T cell pool [28]. In contrast, non‐conventional lineages rely directly on mTEC‐driven signals, and the absence of mTECs, or specific mTEC subsets, results in profound defects in *de novo* Treg and iNKT cell differentiation [28, 57]. This requirement has long been obscured by contamination from recirculating peripheral Tregs, which accumulate in the medulla and further shape the selection landscape [73, 74, 82]. These recirculating populations actively modulate the medullary microenvironment, adding an additional regulatory layer to late‐stage thymocyte maturation [74, 76]. Collectively, these tightly coordinated processes ensure the formation of a robust and self‐tolerant T cell repertoire. A precise and comprehensive understanding of medullary biology is therefore essential, both for identifying how perturbations drive immune pathology and for defining opportunities to strategically manipulate the medullary niche to enhance immune resilience.

## 3 The Thymic Medulla Across a Life Span

Much of this review has focused on how the medulla shapes the development of conventional and non‐conventional T cell subsets, yet an equally important, and often overlooked, consideration is how its structure and functional capacity change across the lifespan. Understanding these age‐related changes in TEC composition and function is essential for determining how the medulla continues to support T cell development throughout life. Thymic function is a key determinant of the host's immunological fitness, yet its activity is not constant across the lifespan. During development, the thymus undergoes a marked increase in size. In humans, it reaches its maximal size within the first year of life [99], while in mice it peaks at approximately four to six weeks of age [100, 101]. Following this early expansion, the thymus begins to atrophy in both species, with involution occurring no later than the onset of puberty. Notably, the thymus is the first organ to undergo age‐associated involution and does so at a faster rate than other tissues [102]. In humans, the thymus progressively shrinks and is gradually replaced by adipose tissue, leading to reduced T cell production and diminished repertoire diversity with age [103]. Similarly, aging in mice is accompanied by a substantial decline in thymic cellularity, resulting in decreased thymic output and fewer RTEs in the periphery [104]. Despite this chronic involution, the adult human thymus retains functional activity, as evidenced by the increased risk of all‐cause mortality and cancer observed following thymectomy in adulthood [105]. In addition to gradual age‐associated decline, the thymus can also undergo acute involution in response to various stressors. Importantly, it retains a remarkable capacity for regeneration after such insults [106].

TECs orchestrate the dynamic changes in thymic size throughout life. TEC number closely correlates with overall thymic size, reflecting their rapid expansion during early development and their decline during aging [100, 107, 108, 109, 110]. Disrupted TEC development in the adult thymus leads to severe thymic atrophy [111, 112, 113], whereas expansion of adult TECs promotes increased lymphocyte development and can result in pronounced thymic hyperplasia [100, 107, 108, 109]. Furthermore, TECs exhibit age‐specific properties, including distinct transcriptional programs [100], developmental trajectories [114], and functional capacities [115, 116]. Understanding the age‐dependent characteristics of TECs provides important insight into the mechanisms that regulate thymic size across the lifespan and, consequently, the immunological fitness of the host.

### 3.1 Unique Properties of Fetal and Adult TECs

The marked expansion of thymic size and TEC abundance during fetal and neonatal life is driven by properties that distinguish early TEC populations from their postnatal counterparts. Fetal TECs exhibit higher proliferative activity [101] and increased ribosomal biogenesis compared with adult TECs [100]. Functionally, they are also distinct: fetal TECs can successfully engraft following intrathymic transfer into adult recipients, a capacity that is lost after birth [116]. In addition, they uniquely support the early waves of γδ T cell development [115]. Differences also emerge within the progenitor compartments of fetal and postnatal TECs. A bipotent TEC progenitor is detectable as early as embryonic day 13.5, capable of generating both cortical and medullary lineages [117, 118]. During early organogenesis, some progenitors transiently express cTEC‐associated markers, including β5t, CD205, and high IL‐7, before committing to the medullary lineage [119, 120, 121]. Progenitors already restricted to an mTEC fate can also be identified [122], including a Keratin 19 expressing multipotent mTEC progenitor found in the fetal thymus [123]. In contrast, precursor–product relationships and self‐renewal potential in the adult thymus remain less clearly defined [114]. Bipotent TEC progenitors persist postnatally, marked either by an α6‐integrin^high^Sca‐1^high^Ly51^low^MHCII^low^ phenotype or a Plet1^+^Ly51^+^MHCII^high^ profile [124, 125]. Recent evidence further suggests that fetal and postnatal TEC developmental pathways diverge, with early bipotent progenitors biased toward a cTEC fate, whereas postnatal bipotent progenitors are preferentially directed toward the mTEC lineage [126]. Similarly, TEC stem cells have been identified in the postnatal human thymus, characterized by broad keratin expression and long‐term multilineage potential [127, 128]. Difficulties in comparing TEC populations across human and mouse studies make it additionally challenging to reach a consensus on pathways of TEC development.

We and others have used transcriptomic approaches to compare the transcriptional dynamics of fetal and adult TECs and revealed mechanisms underlying their functional differences. We identified distinct transcriptional programs that differentiate fetal from adult TECs. Fetal TECs showed enrichment for genes involved in cell cycle regulation, ribosomal biogenesis and other established Myc target pathways. Adult TECs displayed enrichment for regulation of the actin cytoskeleton, the lysosomal and antigen‐presentation pathways, suggestive of an adult TEC specific transcriptional program involved in the maintenance of a functional thymus. Examination of genes highly expressed in fetal TECs demonstrated that Myc and its downstream targets drive the rapid expansion of the thymus from embryonic development through young adulthood. From this insight, we developed a transgenic mouse model with sustained Myc expression in TECs, resulting in adult mice that maintain a fetal‐like growth pattern, increased thymic size, and several fetal‐associated features, including enhanced proliferation, elevated ribosome biogenesis, and restored engraftment capacity. Our findings indicate that the developmental decline in Myc activity from fetal to adult stages regulates the rate of thymic growth and ultimately limits adult thymus size. Although our transcriptomic analysis focused on early life, additional reductions in Myc activity were observed between adult and aged TECs, suggesting that the continued decline in Myc contributes to the progressive reduction in thymic size during aging [100].

**FIGURE 3:**
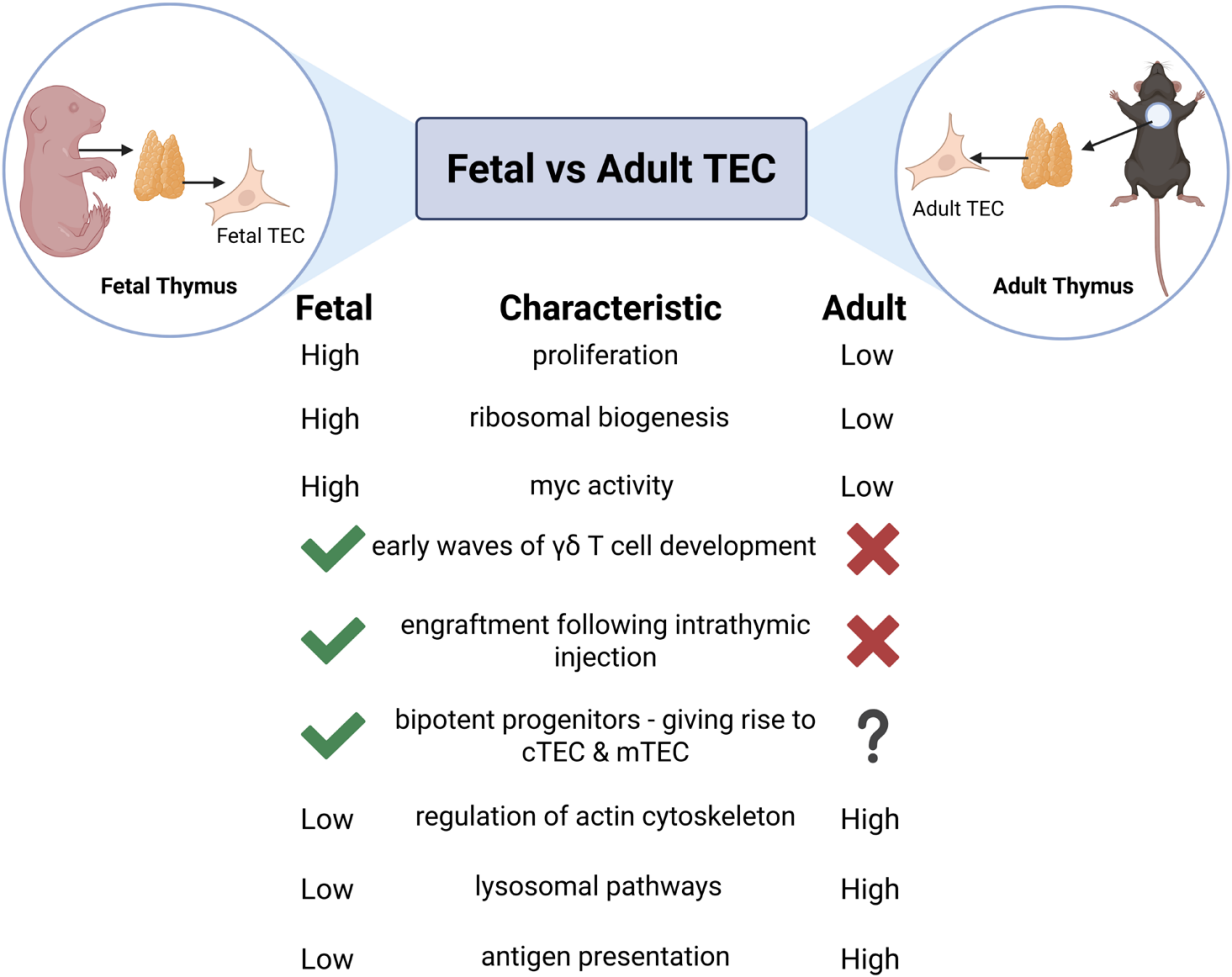
Comparison of fetal and adult thymic epithelial cells properties (TECs). Schematic overview highlighting functional and molecular differences between fetal and adult TECs in the thymus. In the fetal thymus, TECs display high proliferative capacity, elevated ribosomal biogenesis, and increased myc activity. Fetal TECs are associated with early waves of γδ T cell development and show efficient engraftment following intrathymic injection. Evidence also supports the presence of bipotent progenitors capable of giving rise to both cortical TECs (cTECs) and medullary TECs (mTECs). In contrast, TECs in the adult thymus exhibit lower proliferative capacity, low ribosomal biogenesis, and reduced myc activity. The presence of bipotent TEC progenitors in the adult thymus remains unclear. Adult TECs show higher expression of genes associated with actin cytoskeleton regulation, lysosomal function, and antigen presentation. These features illustrate developmental stage‐specific differences in TEC biology and thymic function.

Together, our findings and those of others highlight the distinct properties that distinguish fetal from adult TECs and shape the early thymic microenvironment (summarized in Figure 3). Understanding these differences not only clarifies how the thymus develops but also shows that key fetal programs can be reactivated to enhance growth and restore thymic size later in life. Harnessing these pathways offers powerful opportunities to modulate thymic function across the lifespan [100].

### 3.2 Adult TEC Diversity

Adult TECs exhibit a turnover of approximately two weeks [101], yet their capacity for self‐renewal and the precise relationships between precursor and mature populations remain incompletely defined. This complexity is further compounded by the extensive heterogeneity of TECs in the adult thymus, which has only begun to be revealed through recent advances in single‐cell technologies. As we are still unraveling the functional significance of this heterogeneity, the developmental trajectories that govern TEC differentiation also remain largely unknown [129].

Much of the postnatal TEC diversity identified to date is concentrated within the mTEC compartment, whereas distinct cTEC subpopulations appear comparatively limited [129]. This apparent paucity of cTEC heterogeneity may partly reflect technical challenges in isolating these cells from the adult thymus, owing to their tight physical association with thymocytes [130, 131]. Using conventional enzymatic digestion, only very small numbers of cTECs can be recovered, markedly underrepresenting their true abundance as estimated by imaging based approaches [132].

Despite this limitation, cTECs in the postnatal thymus do exhibit notable heterogeneity [130]. A substantial subset consists of thymic nurse cells which are specialized epithelial cells that envelop and support DP thymocytes during survival and TCRα rearrangement [131, 133, 134]. In addition, adult cTECs display heterogeneity based on expression of the Notch ligand delta‐like ligand 4 (DLL4) [135], a critical factor for commitment of lymphoid progenitors to the T cell lineage [136, 137]. Whereas fetal and neonatal cTECs uniformly express high DLL4 levels, adult cTECs can be subdivided into two major DLL4‐defined subsets with distinct transcriptional profiles [130, 135]. Further cTEC diversity arises from differential expression of CXCL12 [138]. CXCL12 and its receptor CXCR4 mediate the positioning of early T cell progenitors along with TCRβ selection and maturation [139]. CXCL12 positive cTECs are intermittently positioned throughout the adult cortex [140]. Although neonatal cTECs homogeneously express Cxcl12, only approximately 60% of adult cTECs remain Cxcl12‐positive [138]. The Cxcl12 negative subset lacks Foxn1 expression and correspondingly downregulates multiple Foxn1‐dependent genes, including Cxcl12 itself, Dll4, Ccl25, Psmb11, and Prss16, despite originating from Foxn1‐expressing TECs. Differentiation of the Cxcl12 negative population requires signals from DP thymocytes, as their frequency is markedly reduced in Rag2‐deficient mice which have a halt in development at late‐stage, DN. Collectively, these findings indicate that DN‐to‐DP thymocyte maturation plays a key role in shaping cTEC diversity in the adult thymus [138].

Single‐cell RNA‐sequencing confirmed that the cTEC compartment is heterogeneous, with one study identifying two principal subsets: perinatal cTECs, enriched during the perinatal period, and mature cTECs, which predominate in the adult thymus [141]. This subdivision was further validated using single‐cell protein profiling, which identified CD83, CD48, and Sca‐1 as surface markers that reliably distinguish perinatal cTECs from their mature counterparts [142]. The developmental heterogeneity of cTECs and the functional roles of these different subsets, however, remain poorly understood.

In contrast to cTECs, mTECs display striking functional and morphological diversity in the adult thymus [129]. Early studies first defined mTEC heterogeneity based on differential expression of MHC class II and CD80 [101, 143]. Two principal populations were identified. The mTECˡ° subset expresses lower levels of MHC class II and CD80, whereas the mTEC^hi^ subset expresses high levels of both markers and contains the Aire‐expressing cells that are central to the enforcement of self‐tolerance [101]. This population is also enriched for mTEC expressing Fez family zinc finger 2 (Fezf2), another known regulator of a unique set of TRAs in mTECs that are important for establishing central immune tolerance [144, 145]. However, recent studies suggest that Fezf2 may instead play a more indirect role in TRA expression by regulating the development of late‐stage mTECs [146].

The mTECˡ° compartment is functionally and developmentally heterogeneous. It includes CCL21‐producing cells that recruit positively selected SP thymocytes into the medulla [147, 148, 149], as well as cells expressing type 2 cytokine receptors that secrete CCL11, guiding mature thymocytes toward thymic egress [150]. Developmentally, mTECˡ° cells can give rise to mTECʰᶦ subsets, including Aire and Fezf2 expressing mTECs [151, 152, 153]. An intermediate proliferative mTECˡ° population expressing Fezf2 lies upstream of Aire^+^ mTECʰᶦ cells [8, 141]. This subset corresponds to the transit‐amplifying mTEC population termed TAC‐TECs, which generates both CCL21‐expressing and Aire‐expressing progeny [154]. Notably, the mTECˡ° pool also contains terminally differentiated cells that have previously expressed Aire [155, 156], but lack CCL21 expression [157]. Thus, the mTECˡ° compartment encompasses progenitors, intermediates, and post‐Aire cells at distinct stages of differentiation.

Fate‐mapping and single‐cell transcriptomic analyses further revealed previously unrecognized complexity within the mTEC lineage, including a distinct tuft‐like population [62, 130]. Thymic tuft cells share features with mucosal chemosensory tuft cells but represent a specialized epithelial subset that integrates properties of conventional mTECs and peripheral tuft cells [62]. Their development depends on the transcription factor Pou2f3, and they contribute to central tolerance through IL‐25 production while modulating intrathymic iNKT2 cells [62] (described in the section “Invariant Natural Killer T cell differentiation”) and ILC2s [130, 158]. These cells express Doublecortin‐like kinase 1 (DCLK1) and are detectable in the human thymus. Although thymic tuft cells arise from Aire‐expressing mTECs, their generation is preserved in Aire‐deficient mice, indicating an Aire‐independent pathway [130]. In contrast, their formation requires Lymphotoxin β receptor (LTβR), an essential regulator of multiple mTEC subsets [158]. Moreover, a recent study identified a new role for FezF2 as a regulator of thymic tuft cell specific gene signatures that are essential for their development [146].

The identification of thymic tuft cells formed part of a broader recognition of “mimetic” mTEC subtypes that redefine the organization of tissue‐restricted antigen expression within the medulla [159]. These mimetic epithelial subsets adopt peripheral tissue transcriptional and chromatin programs downstream of Aire and rely on peripheral lineage‐defining transcription factors to assemble coherent, tissue‐specific antigen modules. In addition to expanding the antigenic landscape, mimetic subsets such as tuft‐like and microfold‐like mTECs influence medullary function through cytokine and chemokine production and through interactions with innate lymphoid cells, B cells, and developing T cells [160]. The mimetic compartment includes several rare epithelial subsets, including ciliated‐like TECs [161], microfold‐like TECs [160] neural‐like TECs [141] and additional populations such as endocrine‐like and myocyte‐like epithelial cells, identified by multiomic analyses [83]. These studies have begun to assign functional roles for these specialized subsets in tolerance induction and thymic homeostasis [83].

Comprehensive single‐cell atlases of the human thymic stroma have revealed even greater epithelial heterogeneity [162, 163]. Rare subsets include CFTR^+^ ionocytes, ciliated cells, and Schwann‐like cells, alongside lineage trajectories linking neuroendocrine and myoid fates. These analyses also identified stromal populations that shape TEC development, such as Wnt‐producing mesenchymal cells and activin A‐expressing pericytes, and mapped tissue‐restricted antigen expression across subsets, clarifying both Aire‐dependent and Aire‐independent pathways of central tolerance [163]. Importantly, *bona fide* epithelial stem cells have been identified in the postnatal human thymus. These clonogenic, expandable cells reside in both cortical and medullary regions and display multilineage potential to generate cTECs and specialized medullary lineages, including neuroendocrine, ionocyte, and myoid populations. They can reconstitute cortical and medullary organization *ex vivo,* reshaping current understanding of human TEC maintenance and involution [164].

Taken together, studies in mouse and human systems underscore the remarkable heterogeneity, developmental plasticity, and functional specialization of the mTEC compartment. The coordinated interplay among diverse mTEC subsets is indispensable for the establishment and maintenance of central self‐tolerance [165]. Defining how this epithelial heterogeneity is generated, sustained, and functionally integrated across the lifespan is therefore critical to understanding how a self‐tolerant T cell repertoire is continuously shaped in adulthood, and how perturbations in these processes may emerge during thymic aging. The identification of mimetic mTECs in both mouse and human challenges our current understanding of self‐representation in the thymus and heralds an exciting era in unraveling the cellular and molecular mechanisms that orchestrate central tolerance within the highly specialized medullary microenvironment.

### 3.3 TEC Diversity in Aging

With advancing age, the thymus gradually shrinks and its internal structure becomes increasingly disorganized. Distinct cortical and medullary boundaries become less defined, fibroblasts expand, epithelial‐free regions appear, and adipocytes progressively accumulate. Multiple mechanisms contribute to this process, including hormonal alterations and chronic low‐grade inflammation, which together affect both hematopoietic and stromal compartments. Within this changing environment, TECs undergo profound qualitative and quantitative remodeling. While early studies emphasized declining thymic size and cellularity, more recent work demonstrates that aging reshapes TEC composition, progenitor activity, transcriptional identity, and epithelial architecture [110, 166].

Functional decline of the thymus is evident by middle age in mice, around 12 months [167], with deregulation of genes associated with involution detectable as early as 3–6 months [168]. Aged TECs show a decline in cell cycle‐related genes, including diminished E2F3 activity [168], detectable from as early as 2–10 weeks of age [169]. Moreover, a decline in Myc activity, including cell proliferation rates and ribosomal biogenesis is seen between adult to aged TECs [100]. A major advance in understanding TEC aging came from transcriptomic approaches [141, 168] Baran‐Gale and colleagues used single‐cell transcriptomic and lineage‐tracing studies demonstrated that age‐related thymic decline is primarily driven by dysfunction within epithelial progenitor populations rather than intrinsic defects in mature TEC subsets. Aging disrupts progenitor differentiation, including the early loss of a cortical precursor population after puberty and progressive quiescence of a medullary progenitor pool. Impaired progenitor activity compromises maintenance of the epithelial scaffold, reducing the efficiency of T cell selection, altering self‐antigen presentation and TCR diversity. This study positions progenitor attrition as a central mechanism of thymic involution and suggests potential avenues for targeted thymic regeneration [141].

Age‐related changes are not limited to progenitor loss but also involve marked structural remodeling. It has been demonstrated that thymic atrophy is not primarily due to TEC loss or reduced proliferation. Instead, cortical TECs progressively lose their characteristic cellular projections, which are essential for supporting thymocyte interactions. Regeneration correlates with restoration of these structures rather than increased epithelial cell number. The findings further indicate that medullary stromal cells regulate cTEC morphology through paracrine morphogens and growth factors, shifting emphasis from epithelial survival to architectural integrity as a key determinant of thymic size and function [131]. These results complement the finding that gene expression profiles of cTECs display a greater change during the involution process in comparison to mTECs [170].

In addition to progenitor dysfunction and cTEC remodeling, distinct age‐associated TEC states emerge during involution. Using single‐cell and spatial transcriptomics combined with lineage tracing and advanced imaging, Kousa and colleagues identified age‐associated TECs (aaTECs) [171]. These cells form epithelial clusters devoid of thymocytes, increase in frequency with age, and display features consistent with epithelial‐to‐mesenchymal transition. Their accumulation is exacerbated by acute injury and correlates with reduced regenerative capacity. Mechanistically, aaTECs were suggested to sequester key regenerative signals, including Fibroblast Growth Factor (FGF) and Bone Morphogenetic Protein (BMP) family factors, providing a possible explanation of how they might limit trophic support to functional TEC populations [171].

Collectively, these studies redefine TEC diversity in aging as a dynamic process involving progenitor attrition, architectural changes of cTECs, altered stromal crosstalk, transcriptional reprogramming, and the emergence of novel age‐associated epithelial states. Rather than simple epithelial loss, thymic involution reflects coordinated changes in TEC differentiation, morphology, and intercellular signaling that undermine thymic output and immune tolerance.

### 3.4 Immune Consequences of Thymic Involution

Age‐related declines in adaptive immunity have long been recognized, yet the specific impact of reduced thymic size and function on this systemic immunodeficiency remains incompletely defined [172, 173]. Immune senescence in aging is associated with increased susceptibility to infections, poor vaccine response, autoimmune disease, and malignancy [174, 175, 176, 177]. Although thymic involution is widely proposed to contribute to impaired immune competence and heightened infection risk in older individuals, direct experimental evidence establishing this causal relationship is still limited [178]. Part of this knowledge gap stems from a lack of models that restore thymic size in aged mice or humans.

Thymic involution results in a reduction of RTEs in the periphery of aged mice [104]. Although more difficult to assess in humans, TCR excision circles (TRECs), which serve as a marker of newly generated T cells, are reduced in elderly individuals [179, 180], though the precise age at which this decline begins remains unclear [181]. In mice, the age‐associated decrease in RTEs is accompanied by reductions in peripheral naïve T cells [182, 183, 184] and limitations in TCR repertoire diversity [184, 185]. In humans, diminished thymic function correlates with lower naïve T cell counts, particularly affecting naïve CD8 T cells, and is associated with shorter telomeres as well as aberrant activation and proliferation within these cells [186].

Certain viruses show increased severity in aged mice due to age‐related changes in T cell responses. For example, older mice infected with West Nile virus exhibit lower survival rates than younger mice, a vulnerability linked to defects in both CD8 and CD4 T cell‐mediated immunity [187]. Influenza is also influenced by age. In aging individuals, reduced protective immunity against influenza can result from a decline in CD8 T cell repertoire diversity, creating gaps in the repertoire that hinder effective viral control [188]. Additionally, specific strains of the parasite *Toxoplasma gondii* can be lethal in aged mice while causing only mild illness in young mice [189, 190], with T cells playing a pivotal role in this observed age‐associated mortality [184].

In summary, age‐related thymic involution reduces the peripheral T cell pool, leaving older individuals more susceptible to infections. Interventions aimed at restoring thymic function therefore have the potential to confer substantial clinical benefits. Beyond gradual age‐associated shrinkage, the thymus is also highly sensitive to stimuli that induce acute involution [106]. Much of our understanding of thymic regeneration after acute injury comes from studies of hematopoietic stem cell transplantation (HSCT), where impaired T cell recovery is a major contributor to morbidity and mortality [191]. Patients who rapidly reconstitute T cell populations following HSCT are less likely to experience severe graft‐versus‐host disease (GVHD) or relapse and generally have improved overall survival [191]. In murine models, the thymic medulla often fails to regenerate fully after total‐body irradiation [164, 192], accompanied by reduced expression of tissue‐restricted mTECs [152], which can compromise self‐tolerance and increase susceptibility to auto‐GVHD [164, 192]. These observations highlight additional clinical scenarios in which thymic regeneration could enhance immune competence. However, the functional impact of thymic restoration on protective immunity remains unclear, as recent studies in aged mice report variable success in recovering immune function.

### 3.5 Strategies of Thymic Regeneration

Given the significant immunological consequences of age‐associated TEC disruption, many strategies for thymic rejuvenation have focused on targeting these cells. We therefore adopted a TEC‐mediated approach, reactivating developmental growth programs that we identified as drivers of rapid thymic expansion during early life. Because we previously demonstrated that Myc activity in TECs regulates thymic size [100], we explored whether enforced Myc expression could counter age‐associated thymic involution [184]. We generated a transgenic mouse model with TEC specific constitutive overexpression of Myc, which induced pronounced thymic hyperplasia and sustained thymic output into middle age. Moreover, inducible TEC specific Myc overexpression in aged animals restored thymic size to that of young controls. These models enabled us to examine the consequences of either preventing thymic involution into middle age or restoring thymic size in aged animals [184].

Constitutive Myc‐driven expansion prevented the age associated decline in peripheral naïve CD4 and CD8 T cells, increased TCR repertoire diversity, and enhanced B cell mediated responses. In the inducible model, thymic regrowth partially restored naïve T cell numbers and improved repertoire diversity in the periphery. Importantly, both models demonstrated enhanced T cell dependent immunity following challenge with *Toxoplasma gondii*, a parasitic infection with high mortality in aged mice. Middle aged mice with enhanced thymic function achieved 100% survival compared with 50% in age matched wild type controls, and inducible thymic regrowth in aged mice delayed mortality relative to controls. Our findings provide direct functional evidence that preventing or reversing thymic involution restores T cell production and improves protective immunity in aging [184]. Collectively, our work shows that TEC‐intrinsic growth control serves as a tractable and mechanistically defined target for thymic regeneration, conferring immunological protection in the aging host.

Another major target for thymic regeneration is Foxn1, an indispensable transcription factor for fetal TEC development and postnatal thymic maintenance [108, 111, 112, 113, 183, 193, 194]. Foxn1 regulates proliferation and differentiation across both cortical and medullary TEC lineages [195, 196, 197, 198]. Decline in Foxn1 expression is a hallmark of aging and is strongly implicated in thymic involution [111, 112, 113, 194, 199, 200]. Overexpression of Foxn1 in aged mice reversed thymic atrophy [183, 194], prevented age‐related loss of thymocytes and early thymic progenitors, reduced peripheral CD4 memory T cell expansion, and restored naïve CD4 and CD8 T cell compartments [194]. TEC‐specific Foxn1 upregulation in aged mice similarly promoted thymic regeneration, with increased thymocyte production, restoration of naïve T cell numbers, and architectural and transcriptional profiles resembling those of juvenile thymi [183].

Recent work indicated that Foxn1 activity is highly context dependent. Moderate Foxn1 elevation enhanced TEC differentiation without increasing overall thymus size or delaying involution, whereas Foxn1 upregulation in Plet1^+^ TEC progenitors expanded this progenitor pool but induced aberrant medullary marker expression. In Foxn1 deficient settings, this strategy resulted in a small, medullary biased thymus [201]. In parallel, a recombinant CCR9 Foxn1 fusion protein capable of thymic entry following intravenous administration restored TEC function, enhanced thymopoiesis, and increased peripheral T cell numbers in 14‐month‐old mice. These findings suggest that systemic delivery of Foxn1 based biologics may represent a feasible therapeutic approach to age related T cell deficiency [202].

The demonstration that enforced Foxn1 expression can reprogram fibroblasts into fully functional TECs provided proof that a single transcription factor is sufficient to generate an organized thymic microenvironment capable of supporting complete T cell development in vivo [183]. Building on this, subsequent studies showed that Foxn1 induced TECs can generate *de novo* thymic organs, and that engraftment of Foxn1 reprogrammed fibroblasts into the aged thymus promotes architectural regrowth, restores thymopoiesis, improves negative selection, and reduces inflammatory and senescent T cell populations [196]. Together, these studies position Foxn1 mediated restoration of epithelial identity as a central axis of thymic regeneration.

Aging also disrupts the receptor activator of nuclear factor κB, RANK, and RANKL axis within the thymus. Conditional transgenic mouse models demonstrate that endothelial cell cellularity and maturation depend on RANK signaling. Administration of exogenous RANKL to aged mice restored thymic architecture, increased epithelial cellularity, rejuvenated the mTEC compartment with expansion of mTEC^hi^ and Aire^+^ subsets, reduced apoptosis within mTEC^hi^ cells, and restored endothelial abundance and function [203]. Treated mice exhibited improved responses to vaccination and implanted tumors, enhanced homing of T cell progenitors to the thymus, and increased T cell production. Because RANK is expressed by both TECs and endothelial cells, systemic RANKL delivery likely mediates coordinated stromal rejuvenation rather than mTEC specific effects [203].

Pharmacological approaches, including administration of interleukin 7 (IL‐7), keratinocyte growth factor (KGF), and the sex steroid–ablation agent degarelix, have been shown to temporarily increase T cell production [204, 205, 206]. While sex steroid ablation can enhance immune responses, the magnitude of restoration is age‐dependent, with improvements in thymic function after castration diminishing between nine and 18 months. For instance, nine‐month‐old castrated males challenged with Influenza A exhibited virus‐specific cytotoxic CD8 T cell numbers comparable to young mice, accompanied by more efficient viral clearance in the lungs. By contrast, 24‐month‐old castrated mice did not reach young‐level CD8 T cell restoration but still demonstrated reductions in lung viral titers. These observations indicate that age‐related defects in T cell immunity are partially driven by reduced naïve T cell repertoires, and that thymic regeneration through sex steroid ablation can partially correct this deficit and improve viral immune responses, with additional T cell–independent mechanisms contributing to viral clearance [207]. Similarly, KGF treatment and sex steroid ablation increased thymic size and cellularity in aged mice and nonhuman primates, as well as raised levels of RTEs in the blood, although few were detected in the spleen or lymph nodes, likely reflecting age‐associated structural impairments in secondary lymphoid organs. Importantly, sex steroid ablation did not enhance survival following West Nile virus challenge, suggesting that thymic enlargement alone is insufficient to fully overcome peripheral immune limitations that constrain functional T cell rejuvenation [182].

Beyond TEC centred approaches of thymic regeneration, mesenchymal cells are emerging as critical regulators of thymic regeneration. Three distinct thymic mesenchymal cell subsets have been identified in mouse and human thymus [191]. Although their developmental relationships remain unresolved, these stromal populations differ in their capacity to recruit hematopoietic progenitors, a process that becomes limiting with age as early T cell precursors decline. Engraftment of the CCL19^+^ mesenchymal subset increased early thymic progenitors and enhanced mature T cell output in aged mice. The persistence of engrafted cells and the ability to engineer bone marrow derived mesenchymal cells with thymopoietic properties underscore the translational potential of stromal populations capable of reversing key bottlenecks in the aging thymus [191].

Systemic hematopoietic aging also contributes to thymic decline. Age‐associated expansion of myeloid biased hematopoietic stem cells reduced lymphoid progenitor availability. Antibody mediated depletion of myeloid biased stem cells restored common lymphoid progenitors, expanded naïve T and B cell pools, reduced exhaustion markers and inflammatory mediators, and improved antiviral responses [208]. Complementing this, hepatic delivery of mRNAs encoding Delta like ligand 1, FLT3L and IL‐7 expanded common lymphoid progenitors, enhanced *de novo* thymopoiesis, replenished peripheral T cell pools, and improved vaccine and antitumor responses in aged mice [209]. These data illustrated that correcting progenitor supply can synergize with thymus intrinsic repair [209].

Collectively, the aged thymus remains responsive to diverse regenerative interventions. However, the extent to which structural restoration translates into durable immune competence varies considerably. We demonstrated Myc‐driven TEC rejuvenation currently provides a clear demonstration of functional benefit, linking epithelial regrowth to improved T cell repertoire diversity and protection against otherwise lethal *Toxoplasma gondii* infection in aged mice [184]. By contrast, although Foxn1‐based approaches robustly restore epithelial identity and thymic architecture, most studies have not comprehensively assessed downstream pathogen resistance or systemic immune performance [183, 194, 201, 202]. Defining the relationship between epithelial repair, progenitor recruitment, peripheral integration, and protective immunity will be essential for translating thymic regeneration into clinically meaningful immune rejuvenation.

Successful restoration of T cell development will likely require coordinated targeting of multiple components of the thymic niche, combining TEC specific regeneration with modulation of other stromal populations and restoration of competent hematopoietic progenitor pools. Strategies focused exclusively on a single compartment may be insufficient. Such integrated approaches may ultimately re‐establish a functional thymic microenvironment capable of restoring and sustaining a diverse naïve T cell repertoire in aging or after acute thymic injury, thereby providing protection against a broad range of immunological insults.

### 3.6 Mechanisms of Endogenous Thymic Regeneration

Beyond gradual age‐related involution, the thymus undergoes acute atrophy in response to many stressors such as infection, pregnancy, malnutrition, cytotoxic drugs, irradiation, and inflammatory stimuli. However, the thymus retains a notable intrinsic capacity for repair, although this regenerative potential diminishes with advancing age. In experimental models, agents such as cyclophosphamide, lipopolysaccharide, dexamethasone, and ionizing radiation induce profound thymic shrinkage and are used to investigate endogenous mechanisms of thymic regeneration once the insult resolves [210]. This intrinsic regenerative capacity indicates that thymic stroma retains latent repair programs that restore TEC integrity and thymopoiesis. Multiple studies have identified coordinated epithelial, mesenchymal, endothelial, and immune cell circuits that drive this recovery, providing mechanistic insight into how the thymus re‐establishes T cell development after injury [106, 211].

A central axis of endogenous repair involves cytokine mediated restoration of the TEC compartment. IL‐22, produced by ILCs downstream of IL‐23 from CD103^+^ conventional (c)DCs, is dispensable at steady state but becomes essential following sublethal total body irradiation, where it promotes TEC recovery and thymocyte reconstitution [211]. IL‐22 signals through STAT3 to enhance TEC survival and upregulate key regulators including Foxn1, Aire, and Kgf, and exogenous IL‐22 accelerates thymocyte reconstitution and mitigates chronic GVHD [212, 213]. Transcriptomic analysis of thymic stroma identified BMP4 as a damage induced factor enriched at the onset of regeneration. Although expressed by fibroblasts and endothelial cells, only endothelial cells upregulate BMP4 after injury. Endothelial specific deletion of Bmp4 impairs thymic recovery, demonstrating its functional importance. Endothelial derived BMP4 act on TECs to induce Foxn1 and downstream targets including Dll4 and Kitl, with effects largely restricted to cortical TECs rather than medullary mTECs [214]. Damage sensing through immunogenic cell death and inflammasome activation further amplifies repair. Release of DAMPs such as zinc, ATP, IL‐25, and IL‐33 from tuft cells and fibroblasts activates ILC2s, which produce amphiregulin, IL‐5, and IL‐13 to support epithelial differentiation and thymic rebound [215, 216, 217].

Additional immune populations modulate this process. Eosinophils recruited in a CCR3‐dependent manner contributed to epithelial restoration via NKT cell, IL‐4, and ILC2 circuits [218, 219], while recirculating thymic Tregs expanded after injury and promoted repair in an amphiregulin‐dependent manner, with analogous CD39^+^ICOS^+^ Tregs identified in the human thymus [98]. Conversely, IL‐18 generated during caspase 1‐mediated cell death activated NK cells that limited regeneration by targeting TECs, identifying inhibitory pathways that may be therapeutically restrained [220]. The reasons for the involvement of multiple distinct pathways in the same regenerative outcome remain unclear, but the specific circuits engaged may depend on the nature or severity of the insult.

Physiological involution during pregnancy further illustrates the plasticity of TECs. Gestation induced transient thymic shrinkage through progesterone mediated downregulation of Foxn1 and reduced chemokine expression required for progenitor recruitment [221, 222]. Notably, TEC numbers remained stable, and early postpartum regeneration was driven primarily by rapid transcriptional reprogramming of cTECs, including restoration of Foxn1‐dependent networks [221]. RANK linked pregnancy hormones to thymic remodeling by enabling progesterone driven AIRE^+^ mTEC expression. This consequently resulted in Treg expansion during gestation. TEC specific loss of Rank during pregnancy prevented normal thymic involution, reduced placental Treg accumulation, and led to fetal loss and metabolic disturbances resembling gestational diabetes, defects that were reversible by Treg transfer [223]. Finally, studies of irradiation induced injury revealed that incomplete recovery of mature Aire^+^ mTECs and late‐T stage differentiation can persist despite overall thymic rebound, highlighting selective vulnerabilities within the TEC compartment that may predispose to autoimmunity [164].

Together, these convergent injury and physiological models demonstrate that the thymus possesses robust endogenous repair programs centred on TEC plasticity, stromal crosstalk, damage sensing, and immune‐mediated feedback. The same molecular circuits that restore TEC identity and thymopoiesis after acute stress likely represent exploitable pathways for thymic regeneration, offering a blueprint for therapeutic strategies aimed at reactivating intrinsic thymic repair in aging and immune deficiency.

We speculate that thymic involution may represent a context‐dependent, “tailored” remodeling process rather than a purely degenerative decline, potentially explaining why distinct molecular pathways can converge on similar regenerative outcomes. Early evidence suggests that in certain physiological contexts, such as pregnancy [223], thymic involution may produce host‐beneficial outcomes, including enhanced *de novo* regulatory T cell production. These observations raise the possibility that thymic remodeling can serve adaptive functions. At the same time, they highlight the importance of examining models of thymic injury and regeneration individually to understand their specific consequences for T cell development. Such insights may ultimately guide regenerative strategies aimed at directing more precise outcomes in thymic function and T cell differentiation.

### 3.7 Summary

The thymus is a highly dynamic organ whose structure and function are shaped by age‐dependent changes in TECs. Fetal and neonatal TECs drive rapid thymic growth and support early T cell development, whereas adult TECs exhibit limited proliferative capacity, extensive heterogeneity, and specialized medullary subsets essential for central tolerance. Age‐associated involution involves progenitor loss, architectural disruption, and the emergence of functionally altered TEC states, resulting in reduced thymic output, diminished naïve T cell pools, and impaired TCR diversity. Regenerative models, including our TEC‐specific Myc overexpression approach and others such as Foxn1 activation, RANKL signaling, and pharmacological interventions, have demonstrated enhanced thymic regrowth, restoration of T cell development, and increased naïve peripheral T cell numbers and diversity. In some cases, this regeneration is accompanied by improved protection against immunological challenges. These findings underscore the potential to harness thymic plasticity to rejuvenate immune competence in aging and immunodeficient populations.

## 4 Conclusion and Prospectives

The thymic medulla is a cornerstone of immune system integrity, orchestrating T cell fate decisions while adapting throughout life. mTECs provide specialized signals that guide developing thymocytes through effective negative selection, direct lineage commitment of conventional and non‐conventional T cells, and establish a diverse, self‐tolerant repertoire. The thymus undergoes dynamic changes in structure and function, with fetal and neonatal TECs driving rapid thymic growth and early T cell production, whereas adult TECs maintain specialized medullary subsets, exhibit extensive heterogeneity, and preserve central tolerance. Aging gradually disrupts medullary architecture and progenitor activity, leading to reduced thymic output, smaller naïve T cell pools, and contraction of the TCR repertoire. Nevertheless, the medulla retains considerable regenerative capacity, demonstrated both by intrinsic repair mechanisms and interventions that restore TEC growth programs, rejuvenate structural organization, and enhance T cell development. These insights highlight the thymic medulla as both a guardian of T cell identity and a dynamic, adaptable organ whose preservation and restoration are essential for sustaining immune competence from early development through aging.

## Funding

This work was supported by Wellcome Trust and Royal Society, Grant Number 222623/Z/21/Z.

## Conflicts of Interest

The authors declare no conflicts of interest.

## Data Availability

Data sharing is not applicable to this article, as no datasets were generated or analysed during the current study.
